# Ploidy elicits a whole-genome dosage effect: growth of triploid Atlantic salmon is linked to the genetic origin of the second maternal chromosome set

**DOI:** 10.1186/s12863-017-0502-x

**Published:** 2017-04-11

**Authors:** A. C. Harvey, P. G. Fjelldal, M. F. Solberg, T. Hansen, K. A. Glover

**Affiliations:** 1 Institute of Marine Research, P. O. Box 1870, Nordnes, NO-5817 Bergen Norway; 2 Institute of Marine Research (IMR), Matre Research Station, NO-5984 Matredal, Norway; 3 Department of Biology, University of Bergen, P. O. Box 7803, N-5020 Bergen, Norway

**Keywords:** Triploids, Atlantic salmon, Chromosome dosage effect, Growth, Domestication

## Abstract

**Background:**

The Atlantic salmon aquaculture industry is investigating the feasibility of using sterile triploids to mitigate genetic interactions with wild conspecifics, however, studies investigating diploid and triploid performance often show contrasting results. Studies have identified dosage and dosage-compensation effects for gene expression between triploid and diploid salmonids, but no study has investigated how ploidy and parent-origin effects interact on a polygenic trait in divergent lines of Atlantic salmon (i.e. slow growing wild versus fast growing domesticated phenotype). This study utilised two experiments relating to the freshwater growth of diploid and triploid groups of pure wild (0% domesticated genome), pure domesticated (100% domesticated genome), and F1 reciprocal hybrid (33%, 50% or 66% domesticated genome) salmon where triploidy was either artificially induced (experiment 1) or naturally developed/spontaneous (experiment 2).

**Results:**

In both experiments, reciprocal hybrid growth was influenced by the dosage effect of the second maternal chromosome, with growth increasing as ploidy level increased in individuals with a domesticated dam (from 50% to 66% domesticated genome), and the inverse in individuals with a wild dam (from 50% to 33% domesticated genome).

**Conclusions:**

We demonstrate that the combined effect of ploidy and parent-origin on growth, a polygenic trait, is regulated in an additive pattern. Therefore, in order to maximise growth potential, the aquaculture industry should consider placing more emphasis on the breeding value of the dam than the sire when producing triploid families for commercial production.

**Electronic supplementary material:**

The online version of this article (doi:10.1186/s12863-017-0502-x) contains supplementary material, which is available to authorized users.

## Background

Genetic interactions between farmed escaped Atlantic salmon (*Salmo salar* L.) and their wild conspecifics represents one of the main challenges to the expansion of an environmentally sustainable aquaculture industry [1, 2]. Introgression of domesticated salmon in native populations has been documented in many Norwegian rivers, with introgression rates as high as 47% in some cases [3–5]. In order to mitigate this, several methods have been developed, including land-based farming systems and the use of sterile fish [6, 7]. While land-based systems would theoretically eliminate all interactions between farmed and wild salmon, the adoption of this method is hampered by the high running costs and expensive infrastructure [7]. Therefore, triploid salmon, that are sterile, are currently viewed as one of the most promising approaches to inhibit further introgression of farmed escaped salmon in wild populations [8, 9].

As well as inhibiting further genetic interactions with wild populations, farming triploid salmon reduces the incidence of unwanted sexual maturation and its negative effect on growth rate, flesh quality and survival [10], at least in female triploids [11]. However, despite the potential benefits of using sterile triploids for salmon aquaculture, their adoption in commercial production has been delayed by several challenges, including high incidences of skeletal deformities and cataracts [12, 13], increased sensitivity to sub-optimal rearing environments [14], and the inconsistent results found relating to their relative commercial performance compared to diploids, for example growth and survival [6, 8]. The mechanisms underlying these challenges are partially, but not fully known.

In culture, sterile triploid salmon are most commonly produced by a pressure shock causing the retention of the second maternal polar body during meiosis II [6]. The resulting individual has three sets of chromosomes (two from the dam and one from the sire) compared to the normal two chromosome sets of a diploid individual. In the wild, spontaneous triploid individuals have occasionally been observed in several fish species [15], and a recent study found that spontaneous triploidy occurred at an overall frequency of ~2% in Norwegian commercial Atlantic salmon farms in the period 2007–2014 [16]. Polyploidy can occur both within species (autopolyploids) [16] and between species (allopolyploids) [17]. Genome duplication causes several major challenges for cellular processes and important regulatory functions within an organism [18], however autopolyploidy and whole genome duplications are generally not as disruptive as allopolyploidy or partial genome duplication, as overall cellular stoichiometry is maintained [19].

Regulation in response to polyploidisation may occur through gene/genome dosage effects or dosage compensation of regulatory gene complexes that affect gene expression and the resulting phenotype [20]. Dosage effects occur when gene expression correlates with the number of copies of the gene (or chromosome in whole genome duplication), while dosage compensation occurs when genes are expressed at diploid levels even in imbalanced genomes [19]. Mechanisms underlying the consequences of triploidy have been investigated in salmonids through studies which examine gene expression in triploid and diploid full-sibs, indicating positive dosage effects for some genes and dosage compensation for others [20, 21]. Triploid salmonids often display a higher proportion of additive genetic variation, indicating that the effect of an extra chromosome set is additive [22, 23]. However, these studies compared diploid and triploid salmon from the same strain(s), and at present, it is unknown how ploidy will interact with genetics when crossing Atlantic salmon of differing backgrounds. Growth is a polygenic trait and domesticated and wild Atlantic salmon show highly divergent growth under identical farming conditions [24–27]. Therefore, how will an extra maternal chromosome set influence growth when originating from these genetically divergent lines? This question is particularly relevant for breeding programs, as it has been shown in several studies that family performance in various commercially important traits, including growth, may interact with the dosage effect of ploidy [10, 14, 28, 29]. Therefore, it may not be sufficient to select for triploid performance based on diploid families [14, 28, 30, 31], where maternal and paternal contribution to the genome is equal.

Using two complimentary data sets, the present study aimed to investigate the effects of ploidy and parent-origin on the growth of pure wild, pure farmed and reciprocal hybrids of farmed and wild maternal origin in diploid and triploid full and half-sib groups. The first dataset consists of a previously unpublished experiment where triploidy was induced in farmed, wild and reciprocal hybrid Atlantic salmon, while the second dataset consists of a previously published growth experiment [26] where some Atlantic salmon individuals were found to be naturally occurring spontaneous triploids. The combination of the two datasets thus allowed for a unique opportunity to examine the chromosome dosage effect of triploidy on growth in both induced and spontaneous triploids, and whether there is any influence of the genetic origin of the extra maternal chromosome set.

## Methods

### Family production & experimental design

#### Experiment 1

Experimental families were created on 23 November 2011 at the Matre Research Station, Institute of Marine Research (IMR), Norway. One domesticated dam and one domesticated sire originating from the commercial Mowi strain were crossed with one wild dam and one wild sire from the River Figgjo (58°81’N, 5°55’E). The wild parents were caught by angling and transferred to a local hatchery, after which they were transported to Matre to be stripped. Four families were produced: one wild, one domesticated, one maternal-domesticated hybrid cross (domesticated ♀ x wild ♂) and one maternal-wild hybrid cross (wild ♀ x domesticated ♂). Thirty-seven minutes and 30 s after fertilization at 8 °C, half of the eggs from each of the four families were subjected to a hydrostatic pressure of 655 bar for 6 min and 15 s [12]. This resulted in eight experimental groups consisting of four diploid and four triploid families of roughly 150 individuals each. These are hereafter referred to as the experimental groups.

The offspring of each experimental group were hatched and reared in single-strain covered tanks (1 × 1 × 0.25 m) until November 2012. On 26 November 2012, 1134 individuals were sampled for biological measurements (length and weight), from here on referred to as Phase I. Immediately after the completion of Phase I, sixty randomly-selected individuals from each of the eight experimental groups were tagged with passive integrated transponders (PIT-tags) and mixed together into three common garden replicate covered tanks (20 individuals per group per tank = 160 individuals per tank, 480 in total) (1.5 × 1.5 × 0.45 m). The fish were reared under standard hatchery conditions until experiment termination (from here on referred to as Phase II). Therefore Phase I refers to analyses conducted on all the fish from hatch until first sampling in November 2012 (1134 individuals), while Phase II refers to analyses conducted on a subset of fish sampled in November 2012 (Phase I) which were PIT tagged and then allowed to grow until sampling in June 2013 (480 individuals). See Additional file 1: Figure S1 for an overview of the experimental design. Water flow rate was adjusted to ensure oxygen at the outlet was above 80%, water temperature was maintained around 12 °C from first feeding until summer solstice, thereafter ambient water temperature (average: 8.1 °C; range 4.2–14 °C). The photoperiod was LD24:0 from first feeding to 01 October 2012, thereafter simulated natural (61 ^o^N).

The experiment was terminated on 10 June 2013 when the remaining fish (from the 480 individuals) were sampled for biological measurements (length and weight) (end of Phase II). Erythrocyte measurements confirmed the ploidy status of the individuals at this stage. The average weight in each sampling phase and the percentage of domesticated genome in each group is given in Table 1.

**Table 1:** Growth data for experiment 1 and experiment 2

			Diploid					Triploid				
Experiment	Group	Families N	% Domesticated Genome	Final N	Weight (+/− SE)	Length (+/−SE)	CF	% Domesticated Genome	Final N	Weight (+/− SE)	Length (+/−SE)	CF
1: Phase I	Wild	1	0	144	47.80 (1.34)	15.76 (0.19)	1.16	0	134	49.52 (1.43)	16.30 (0.18)	1.10
(all)	Wild x Domesticated	1	50	143	75.94 (1.62)	18.24 (0.14)	1.23	33.3	138	59.77 (1.58)	17.15 (0.18)	1.14
	Domesticated x Wild	1	50	148	64.43 (1.11)	17.44 (0.12)	1.20	66.6	138	82.51 (1.28)	18.88 (0.11)	1.22
	Domesticated	1	100	145	105.70 (2.62)	19.78 (0.15)	1.33	100	144	92.66 (1.42)	19.36 (0.10)	1.26
1: Phase II	Wild	1	0	59	54.44 (1.35)	16.71 (0.13)	1.15	0	60	57.63 (1.62)	17.26 (0.14)	1.10
(PIT tag)	Wild x Domesticated	1	50	59	80.63 (2.54)	18.66 (0.20)	1.22	33.3	60	66.10 (2.23)	17.77 (0.18)	1.15
(beginning)	Domesticated x Wild	1	50	58	69.13 (1.55)	17.78 (0.14)	1.22	66.6	59	83.15 (1.43)	18.93 (0.10)	1.22
	Domesticated	1	100	60	109.17 (3.58)	20.03 (0.19)	1.33	100	59	98.15 (2.13)	19.63 (0.13)	1.28
1: Phase II	Wild	1	0	59	96.93 (3.42)	20.55 (0.17)	1.10	0	60	95.17 (3.60)	20.96 (0.17)	1.01
(PIT tag)	Wild x Domesticated	1	50	59	138.46 (4.83)	23.25 (0.26)	1.10	33.3	60	112.87 (3.87)	22.04 (0.24)	1.03
(end)	Domesticated x Wild	1	50	58	143.02 (4.29)	23.22 (0.18)	1.12	66.6	59	167.53 (3.80)	24.35 (0.15)	1.15
	Domesticated	1	100	60	198.23 (5.76)	25.73 (0.24)	1.15	100	59	185.86 (4.79)	25.27 (0.20)	1.14
2	Domesticated x Wild	5	50	175	22.96 (0.56)	12.01 (0.12)	1.27	66.6	9	28.33 (2.28)	13.10 (0.34)	1.25
	Domesticated	7	100	233	36.29 (0.52)	13.86 (0.08)	1.34	100	19	30.95 (3.01)	12.97 (0.54)	1.30

Phase I: (all 1134 individuals), Phase II (474 PIT tagged individuals) in experiment 1. Hybrid key: maternal x paternal; N*:* number of families or final number of fish in each group (including outliers where applicable); Weight (mass in grams); Length (fork length); CF condition factor; SE (standard error); Percentage domesticated genome: the relative percentage of the individual’s genome coming from a domesticated parent

#### Experiment 2

The second data set is based upon an analysis of spontaneously-produced triploid fish that occurred naturally in an experiment investigating growth of domesticated, hybrid and wild families [26]. In that study, the low number of spontaneously-produced triploid fish were not specifically investigated. Comprehensive details of the family design and experiment conditions of the second dataset can be found in [26]. The experimental crosses consisted of domesticated, wild and hybrid (domesticated ♀ x wild ♂) families (Additional file 1: Table S2). The parents originated from the commercial Mowi strain and the wild Etne population (59°40’N, 5°56’E). Of the 2256 individuals genotyped in the original study, 71 individuals were identified as trisomic at one or more loci (i.e., triploids) using a microsatellite multiplex. The original experiment entailed a control and a “stress” treatment. Here, we only used data from the control replicates (*N* = 435) which included standard rearing conditions similar to those used in experiment 1. In the control data, there were no wild triploids observed, therefore this dataset contains only domesticated and hybrid individuals. The average weight and the percentage of domesticated genome in each group is given in Table 1.

### Statistical analysis

#### Experiment 1 – Phase I weight analysis including all individuals

Differences in the weight of all individuals were examined using an ANOVA with ploidy and group (and their two-way interaction) as categorical variables. The model’s fit was confirmed by plotting the model residuals against the fitted values and model covariates, and by examining a histogram of the model residuals. Post hoc multiple comparisons were carried out using the *TukeyHSD* function on the full ANOVA in R with a Tukey adjustment for multiple comparisons.

#### Experiment 1 – weight analysis including the PIT tagged individuals at the beginning and end of Phase II

Differences in the weight of the PIT tagged individuals at the start and termination of Phase II were each examined using an ANOVA with ploidy and group (and their two-way interaction) as categorical variables. Where there were replicate tanks (i.e. for the final sampling of Phase II), tank was included in the ANOVA as a random effect. Each model’s fit was confirmed by plotting the model residuals against the fitted values and model covariates, and by examining a histogram of the model residuals. Post hoc multiple comparisons were carried out using the *TukeyHSD* function on the full ANOVA in R with a Tukey adjustment for multiple comparisons.

#### Experiment 2- spontaneous triploidy

Genetic groups (hybrid and domesticated) were analysed separately. Due to the unbalanced nature of the data, the difference in weight of each group was examined using linear mixed models with ploidy as a categorical variable, and family and tank as random effects to control for family and tank variation, respectively. The significance of the fixed effect of ploidy was established by using the *drop1* function based on AIC values [32]. The significance of the random effects were investigated using likelihood ratio tests. The model fit of the chosen model was confirmed by plotting the model residuals against the fitted values and model covariates, and by examining a histogram of the model residuals.

## Results

### The data

#### Experiment 1

At the end of Phase I growth measurements were taken from 1134 individuals. Mortality was very low at the end of Phase II, 477 out of the initial 480 individuals were sampled. Three individuals were missing weight data or had PIT tagging errors and these individuals were removed from the dataset prior to analysis, leaving 474 individuals for data analysis for Phase II. During analysis of final weight three individuals were identified as outliers due to extreme condition factors (>2.0) resulting from possible sampling recording errors and the final weight analyses were repeated without these outliers, therefore this dataset consisted of 471 individuals.

#### Experiment 2

For the present study, only the triploid individuals identified in the control treatment (*n* = 28) were used, together with their full-sib diploid siblings (*n* = 407). The final dataset contained 435 individuals of domesticated (*n* = 251) and hybrid (*n* = 184) origin.

### Statistical analysis

#### Experiment 1 – Phase I

**Fig. 1:**
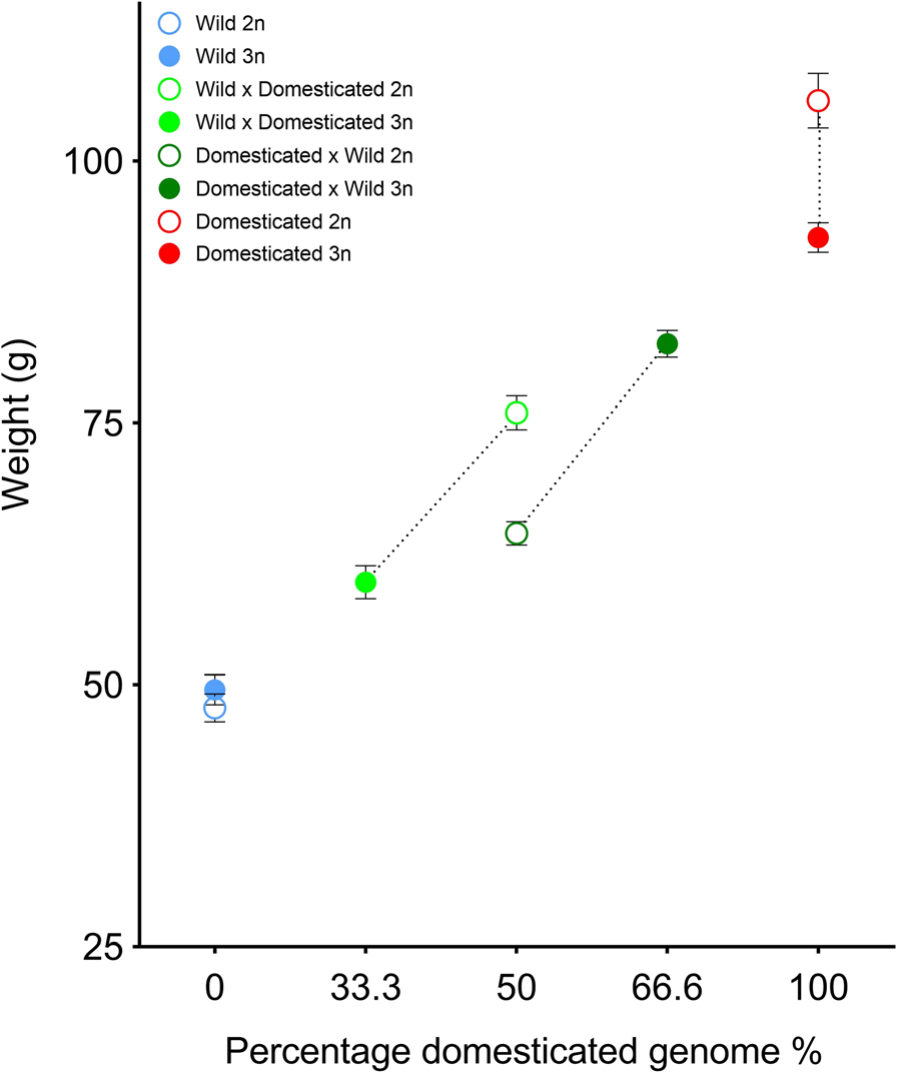
Average weight (g) and standard deviation plotted against the increasing percentage of farmed genome in each Atlantic salmon group for all individuals at the end of Phase I of experiment 1. Diploids are represented by open circles while triploids are represented by closed circles. The stippled black lines indicate full-sib connections

Overall, group had a significant effect on weight at the end of Phase I: For all fish, the domesticated individuals weighed significantly more than the wild origin individuals, and hybrids were generally of intermediate weight (Table 2A, Fig. 1). The overall effect of ploidy alone was not significant, however, a highly significant interaction between ploidy and group was reported for weight (Table 2A).

**Table 2:** Anova outputs of the full models investigating weight

A: Phase I (all) weight	Fixed effects:	Df	Sum sq	Mean sq	F value	P values
	Ploidy	1	1081	1081	3.199	0.074
	**Group**	**3**	**370797**	**123599**	**365.729**	**2E-16**
	**Ploidy x Group**	**3**	**52650**	**17550**	**51.93**	**2E-16**
	Residuals	1126	380534	338		
B: Phase II (PIT tagged) weight beginning)	**Fixed effects:**	**Df**	**Sum sq**	**Mean sq**	**F value**	**P value**
	Ploidy	1	677	677	2.399	0.122
	**Group**	**3**	**138653**	**46218**	**163.776**	**2E-16**
	**Ploidy x Group**	**3**	**15396**	**5132**	**18.186**	**3.6E-11**
	Residuals	464	130940	282		
C: Phase II (PIT tagged) weight end)	**Random effects:**	**Df**	**Sum sq**	**Mean sq**	**F value**	**P value**
	Tank	1	16.3	16.3	0.011	0.932
	Residuals	1	1415.9	1415.9		
	**Fixed effects:**					
	Ploidy	1	2237	2237	1.983	0.16
	**Group**	**3**	**602737**	**200912**	**178.119**	**2E-16**
	**Ploidy x Group**	**3**	**39852**	**13284**	**11.777**	**1.9E-07**
	Residuals	464	523377	1128		

A – Phase I all individuals in experiment 1; B – Phase II (beginning) PIT tagged individuals in experiment 1; and C – Phase II (end) PIT tagged individuals in experiment 1. Df; degrees of freedom, Sum sq; sum of squares, Mean sq; mean squares. Significant variables are highlighted in bold

There was no significant difference detected in weight between wild origin diploids and wild origin triploids (both 0% domesticated genome), while domesticated diploids were significantly larger than domesticated triploids (both 100% domesticated genome) (Table 3A, Fig. 1). For the reciprocal hybrids, weight at the end of Phase I was strongly influenced by the ploidy status and the genetic origin of the parental groups. Diploid hybrids of wild maternal origin (50% paternally-inherited domesticated genome) were significantly larger than their triploid full-sibs (33.3% paternally-inherited domesticated genome), while concurrently triploid hybrids of domesticated maternal origin (66.6% maternally-inherited domesticated genome) were significantly larger than their diploid full-sibs (50% maternally-inherited domesticated genome) (Fig. 1). There was no significant difference in weight between triploid hybrids of domesticated maternal origin and diploid hybrids of wild maternal origin, nor between triploid hybrids of wild maternal origin and diploid hybrids of domesticated origin (Table 3A, Fig. 1).

**Table 3:** Tukey adjusted multiple comparisons of weight between ploidy and groups

A	Wild 2n	Wild 3n	Wild x Dom. 2n	Wild x Dom. 3n	Dom. x Wild 2n	Dom. x Wild 3n	Dom.2n	Dom.3n
Percentage domesticated genome (%)	0	0	50	33.3	50	66.6	100	100
Weight (g)	47.8	49.52	75.94	59.77	64.43	82.51	105.7	92.66
Wild 2n	-							
Wild 3n	NS	-						
Wild x Domestic 2n	***	***	-					
Wild x Domestic 3n	***	***	***	-				
Domestic x Wild 2n	***	***	***	NS	-			
Domestic x Wild 3n	***	***	NS	***	***	-		
Domestic 2n	***	***	***	***	***	***	-	
Domestic 3n	***	***	***	***	***	***	***	-
B	Wild 2n	Wild 3n	Wild x Dom. 2n	Wild x Dom. 3n	Dom. x Wild 2n	Dom. x Wild 3n	Dom.2n	Dom.3n
Percentage domesticated genome (%)	0	0	50	33.3	50	66.6	100	100
Weight (g)	54.44	57.63	80.63	66.1	69.13	83.15	109.2	98.15
Wild 2n	-							
Wild 3n	NS	-						
Wild x Domestic 2n	***	***	-					
Wild x Domestic 3n	***	NS	***	-				
Domestic x Wild 2n	***	***	***	NS	-			
Domestic x Wild 3n	***	***	NS	***	***	-		
Domestic 2n	***	***	***	***	***	***	-	
Domestic 3n	***	***	***	***	***	***	*	-
C	Wild 2n	Wild 3n	Wild x Dom. 2n	Wild x Dom. 3n	Dom. x Wild 2n	Dom. x Wild 3n	Dom.2n	Dom.3n
Percentage domesticated genome (%)	0	0	50	33.3	50	66.6	100	100
Weight (g)	96.93	95.17	138.46	112.87	143.02	167.53	198.2	185.9
Wild 2n	-							
Wild 3n	NS	-						
Wild x Domestic 2n	***	***	-					
Wild x Domestic 3n	NS	NS	***	-				
Domestic x Wild 2n	***	***	NS	***	-			
Domestic x Wild 3n	***	***	***	***	***	-		
Domestic 2n	***	***	***	***	***	***	-	
Domestic 3n	***	***	***	***	***	NS	NS	-

A – Phase I all individuals in experiment 1; B – Phase II (beginning) PIT tagged individuals in experiment 1; and C – Phase II (end) PIT tagged individuals in experiment 1. Percentage domesticated genome: the relative percentage of the individual’s genome coming from a domesticated parent. 2n; diploid, 3n; triploid, p value >0.05: NS; not significant; ***; *p* value < 0, **; *p* value <0.001, *; *p* value < 0.01. Dom.; domestic

#### Experiment 1 – Phase II

**Fig. 2:**
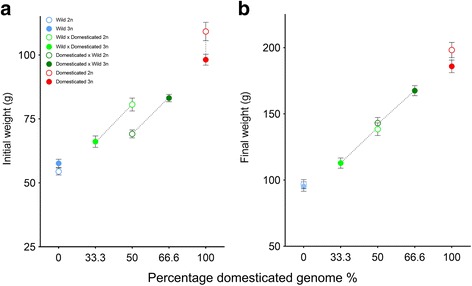
Average weight and standard deviation of PIT tagged individuals (**a**) at the beginning of Phase II and (**b**) the end of Phase II of experiment 1 plotted against the increasing percentage of domesticated genome in each group. Diploids are represented by open circles while triploids are represented by closed circles. The stippled black lines indicate full-sib connections

Overall, group had a significant effect on weight throughout Phase II: domesticated individuals weighed significantly more than the wild origin individuals, and hybrids were generally of intermediate weight (Table 2B, C, Fig. 2a,b). As for Phase I, ploidy alone did not significantly influence weight, but a highly significant interaction between ploidy and group was detected (Table 2B, C).

Throughout Phase II there was no significant difference detected in weight between wild origin diploids and wild origin triploids (both 0% domesticated genome) (Table 3B, C, Fig. 2a, b). At the beginning of Phase II domesticated diploids were significantly larger than domesticated triploids (both 100% domesticated genome) (Table 3B, Fig. 2a), while at the end of Phase II growth between domesticated diploids and triploids (both 100% domesticated genome) did not significantly differ (Table 3C, Fig. 2b).

As above, throughout Phase II, diploid hybrids of wild maternal origin (50% paternally-inherited domesticated genome) were significantly larger than their triploid full-sibs (33.3% paternally-inherited domesticated genome), while concurrently triploid hybrids of domesticated maternal origin (66.6% maternally-inherited domesticated genome) were significantly larger than their diploid full-sibs (50% maternally-inherited domesticated genome) (Table 3B, C, Fig. 2a, b).

When the outliers were removed, the weight of the triploid hybrids of wild origin at the end of Phase II was significantly higher than the wild origin groups (results not presented here but summary data is presented in Additional file 1: Table S1). Tank had no effect in any of the analyses.

#### Experiment 2

**Fig. 3:**
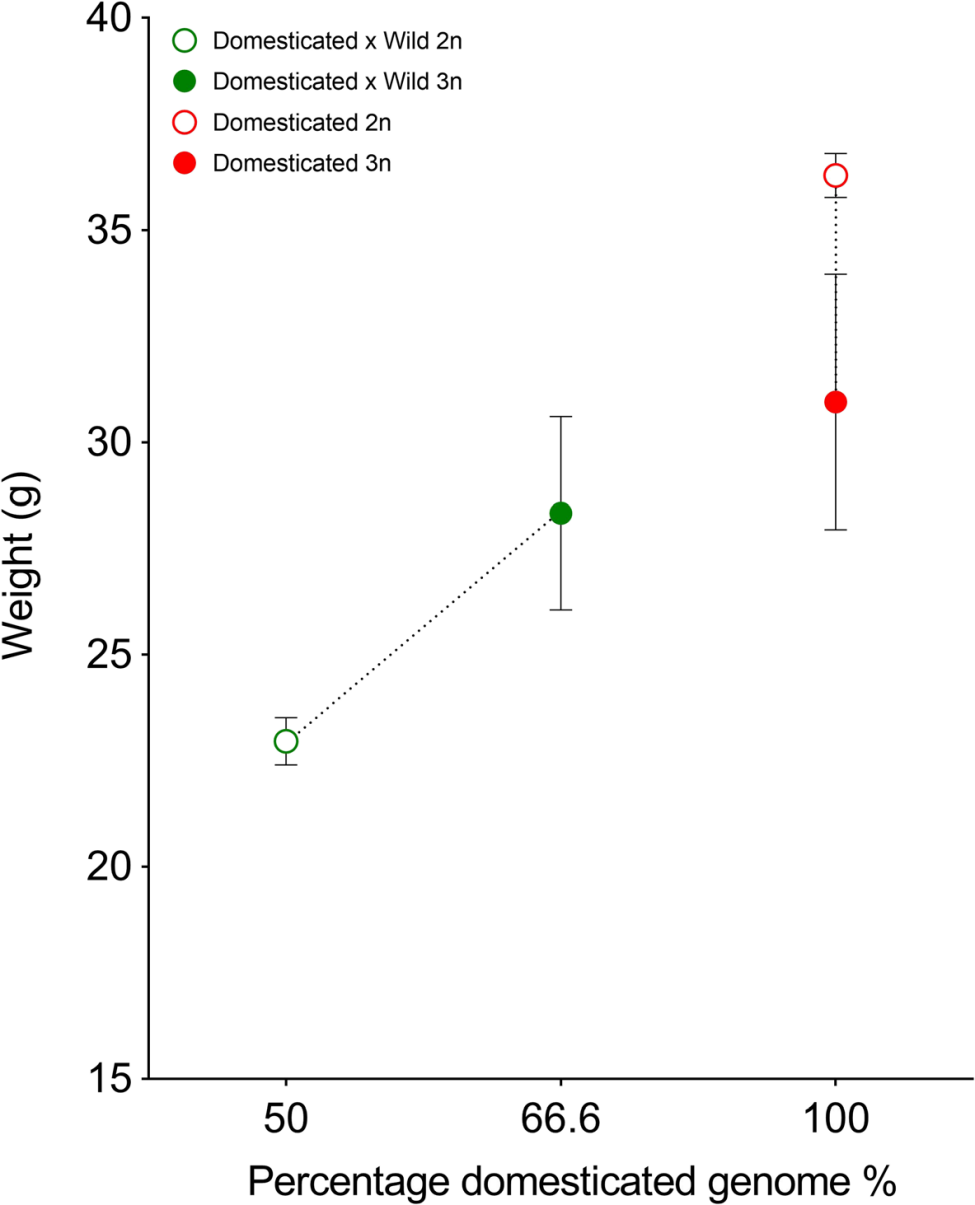
Average weight (g) and standard deviation plotted against the increasing percentage of domesticated genome in each Atlantic salmon group for experiment 2. Diploids are represented by open circles while triploids are represented by closed circles. The stippled black lines indicate full-sib connections

**Fig. 4:**
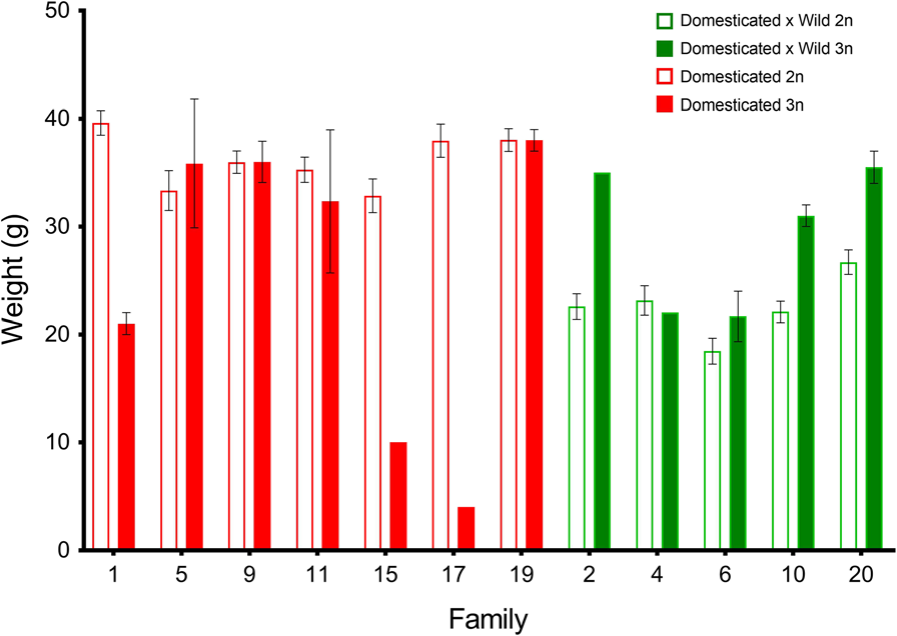
Average weight and SE for each family in experiment 2. Diploids families are the clear bars, and triploid families are the coloured bars. Domesticated families are in red, and domesticated-maternal hybrids are in green

Ploidy had a significant effect on final weight in both groups (Table 4A, B). The average weight of the domesticated individuals (100% domesticated genome) was 36.29 g in diploids and 30.95 g in triploids, while the average weight of hybrid individuals was 22.96 g in diploids (50% maternally-inherited domesticated genome) and 28.33 g in triploids (66.6% maternally-inherited domesticated genome). Therefore, the average effect of ploidy influenced weight in opposite directions for the groups: negatively for pure domesticated triploids and positively for the triploid hybrids (Fig. 3). There was visible variation in weight among the families in both ploidies (Fig. 4). Tank had no effect in either analysis, while family was retained as a random effect in both models (Additional file 1: Table S3). There was a clear trend in Fig. 4 of triploid hybrid families performing either better than or similar to their diploid full-sibs, while the domesticated triploid families performed similar or worse than their diploid full-sibs.

**Table 4:** Model selection of the fixed effect of ploidy in the linear mixed models

**A: Hybrid**		**Fixed effects**	**Random effects**		
N	Response	Ploidy	Family	AIC	ΔAIC
184	Weight	**x**	**x**	**1248.5**	**0**
			x	1253.1	4.6
**B: Domesticated**		**Fixed effects**	**Random effects**		
N	Response	Ploidy	Family	AIC	ΔAIC
251	Weight	x	x	**1785.4**	**0**
			x	1790.4	5

A – the hybrids; and B – the domesticated individuals in experiment 2. AIC; Akaike information criterion. Δ AIC; difference in AIC value. The final fixed effect structure is shown in bold

## Discussion

This is the first study to investigate the potential interaction between ploidy and a polygenic trait when fish originating from highly divergent lines (wild slow growing and domesticated fast growing) are crossed. Results from the two complimentary experiments were consistent, demonstrating that while diploid hybrids tended to display intermediate or close to intermediate growth to their domesticated and wild strains of origin, triploid reciprocal hybrids displayed growth patterns that reflect the dose effect of the second maternal set of chromosomes. I.e., triploid hybrids with 2n domesticated and 1n wild (66% domesticated) and triploid hybrids with 2n wild and 1n domesticated (33% domesticated) grew according to their percent of domestication across the three sets of chromosomes. As growth is a polygenic trait, thus reflecting the combined interactions across large parts of the genome, we conclude that triploid salmon utilise all three chromosome sets, and that this appears to reflect a simple additive, or dosage effect, pattern.

### Dosage effect of triploidy

Triploid salmon are morphologically similar to diploid salmon, apart from sterility and some intracellular differences [21, 33]. All teleost fish share at least three rounds of whole genome duplication (WGD); salmonids have undergone a fourth ancestral tetraploidisation event and the process of re-diploidisation is still on-going [34, 35]. Therefore, salmon have a highly plastic genome, and can withstand the chromosomal changes and genome duplication associated with triploidy [21]. In the present study, triploidy had either a negative or no effect on growth for the pure wild and pure domesticated groups. In experiment 1 triploidy had no effect on growth in the wild group (0% domesticated genome) at Phase I and Phase II (approximately 8 and 14 months after start feeding), while there was a negative effect on growth in the domesticated group (100% domesticated genome) in Phase I, and no significant difference in weight in Phase II. The negative effect of triploidy on growth of individuals with 100% domesticated genome was also observed in experiment 2 (Figs. 3 and 4) (approximately 6 months after start feeding). In salmonids, triploidy has variable effects on growth compared to diploid conspecifics. Several studies limited to commercial strains have shown that triploids show reduced [36, 37] or equal growth to diploids [14, 38], however, studies have also shown that triploids can grow better than diploids [39, 40], although this appears to be life-stage specific [6, 41]. Environmental factors may also influence the performance of triploids, as it is often shown that they do not perform well in sub-optimal conditions [14]. In the present study, it seems that increased ploidy in a pure domesticated (100% farm genome) individual may cause a significant decrease in growth, at least in certain stages, while ploidy has no effect on growth in wild salmon.

There are a lack of studies comparing the growth of diploid and triploid wild origin salmonid strains. Similarly, there are a lack of studies which compare the growth of wild and domesticated diploid or triploid fish relative to each other. However a study by Scott *et al*. [42] found no effect of ploidy on the growth rate of either wild or domesticated rainbow trout (*Oncorhynchus mykiss*) strains. Sacobie *et a.l* [43] compared a diploid wild salmon population to diploid and triploid commercial strains. In contrast to the present study, they found that the weight of a diploid wild Canadian salmon strain was significantly higher than both diploids and triploids of the Mowi strain after 12 weeks in seawater tanks [43]. This result is in stark contrast to several previous studies that compared growth between wild and domesticated diploid salmon [24–26, 44]. It is possible that the contrasting results are due to different wild strains (Norwegian versus Canadian) and differences in selection regimes of the Mowi strain (European versus Canadian).

Diploid salmonid hybrids between two divergent lines (such as wild and domesticated origin) typically display additive/intermediate trait values relative to their parental lines [24, 26, 45, 46]. To date, there is a lack of studies investigating the effects of ploidy on reciprocal half-sibs or hybrids between divergent lines in salmonids. In the present study, although there was a trend of intermediate overall growth of all the hybrid groups, a strong interaction between dosage effect of ploidy and the parent origin caused growth of the triploid hybrids to diverge relative to their full sibs. The growth of the hybrids was regulated by the dosage effect of the second maternal chromosome, with growth increasing as ploidy number increased in individuals with a domesticated dam (from 50% to 66% domesticated genome), and the inverse in individuals with a wild dam (from 50% to 33% domesticated genome). The interaction between ploidy and parent-of-origin on hybrids became more additive over time, with diploid reciprocal hybrids displaying intermediate growth relative to their pure parental strains, and reciprocal triploids growing closer to their maternal-parent strains at the end of Phase II of experiment 1 (Fig. 2). Thus, it appears that the effect of ploidy and parent-origin on growth, a polygenic trait, is regulated in an additive pattern.

Shrimpton *et al*. [20] investigated growth and gene expression differences in triploid and diploid Chinook salmon (*Oncorhynchus tshawytscha*)*.* While growth was lower in triploids, they found no difference in gene expression levels between ploidies, and suggest that positive dosage effects within the cells of the triploids regulates the concentration of regulatory factors involved in gene expression [20]. Ching *et al.* [21] investigated the effect of ploidy on expression levels of specific and genome-wide genes after an immune challenge in Chinook salmon*.* While they found no difference in the gene expression of most of the genes between ploidies, there were significant differences in the expression of some genes relating to immune function [21]. Additive dosage patterns have also been found in allopolyploid hybrids of other organisms. For example, the Australian gecko (*Heteronotia binoei*), a triploid parthenogenetic species complex formed by the hybridisation between two sexual linages that results in four possible cytonuclear combinations (reciprocals with a double chromosome dose from either parent), displays additive genome dosage where offspring with a higher chromosome dosage from one parent were more similar to that parent for various physiological traits [47]. Similarly, differences in the mating call of the triploid interspecific hybrid water frog (*Pelophylax esculentus*) and its parental species *P. lessonae* and *P. ridibundus*, displayed an additive dosage effect depending on the chromosome dosage ratio from the parents [48]. Similar patterns of parent-of-origin or additive genome dosage have been observed in various plant species and species-hybrids [49–52]. While it is intuitive to expect that a phenotypic expression in a triploid hybrid will favour the maternal species, some studies of allopolyploid triploids have shown a mosaic genetic contribution effect on certain phenotypes [17, 53]. To our knowledge, this is the first study to observed additive genome dosage effects in divergent lines of triploid salmon.

### Implications for breeding programs aimed towards commercial triploid production

Several studies have compared the performance of diploid and triploid salmonid full-sib families and found that families ranked consistently among the ploidies [22, 29, 37, 54]. In contrast, other studies have found significant family effects on the performance among diploids and triploids [28, 30, 31], indicating that selection based on diploid full-sibs may not be sufficient to accurately predict triploid performance. In the present study, it was demonstrated that the growth of the triploid hybrids follows a dosage effect of the maternal component. In experiment 2, where more families were examined, the performance of the triploid families relative to their diploid full-sibs appeared to depend on the amount of domesticated genome, with hybrid triploid families performing better or similar to their diploid full sibs, while several domesticated triploid families performed worse than their diploid full-sibs. In most comparative studies between triploid and diploid full sib families, maternal variance of the triploids was larger than for the diploids [10, 22, 55], although see [23].

Blanc *et al.* [55] suggested that family-ploidy effects may be caused by the variation in maternal influence on growth due to the additional genetic maternal provided by the dam. The fact that we observed a dosage-effect of the second maternal chromosome set on growth supports the suggestion by Blanc *et al.* [55]. We suggest that it is this dosage effect which is contributing the conflicting results observed for family ranking between diploid and triploid families in previous studies [22, 28–31, 37, 54]. When the trait performance between the dam and sire is unbalanced, the resulting performance of the triploid offspring will be skewed towards the performance of the dam, as she is contributing more (66% Dam vs. 33% Sire) to the overall performance of the offspring (See Table 5 for a hypothetical example). This does not manifest in diploid offspring as the contributions from both parents are equal (50% Dam vs. 50% Sire). Thus, performance of triploid families relative their diploid full-sib families will vary from study to study due to differences in the performance balance between the dams and sires used. Our results therefore support the suggestion of others [22, 29, 37, 54] that selection based on diploid families may adequate for breeding programs aimed at triploid production. Furthermore, our results suggest that in the final step of the commercial process, when producing triploid families to be reared on farms for food production, then greater emphasis should be placed on the performance of the females compared to the males in order to select the parents to optimise the growth potential of their 3n offspring. Nevertheless, it is still prudent to follow the performance of triploid families with regards to other traits of commercial interest, including disease resistance and stress, as the dosage effect may not be as clear in these contexts. Further studies which investigate the parent-of-origin effect on triploidy in Atlantic salmon under different environmental conditions, and considering other phenotypic traits that are of interest in commercial breeding programs are therefore encouraged.

**Table 5:** Hypothetical example of the dosage effect in terms of the breeding values of the parental lines for three different scenarios relating to the balance of parental performance

	Scenario 1	Scenario 2	Scenario 3
Breeding pair	♀ × ♂	♀ × ♂	♀ × ♂
Breeding value^a^	1 3	2 2	3 1
2n offspring breeding value^b^	2	2	2
3n offspring breeding value^c^	1.66	~2	2.33

Scenario 1 where the dam performance is lower than the sire performance; Scenario 2 where parent performance is equal; and Scenario 3 where the dam performance is superior to the sire

^a^ The breeding value here is a hypothetical value given to the dam (♀) and sire (♂) to represent their growth performance, where 1 < 2 < 3, such that 1 would be a poor performing individual, 2 would be an average performance, and 3 would be a superior preforming individual

^b^ This value represents the growth performance of a diploid offspring where 50% comes from the dam and 50% comes from the sire

^c^ This value represents the growth performance of a triploid offspring where 66% comes from the dam and 33% comes from the sire

### Atlantic salmon as a model species for dosage compensation?

Most studies investigating genome-dosage effects in polyploids focus on differences in gene expression for a selection of genes. Here, we demonstrate a whole-genome dosage effect on a polygenic trait. Several studies have observed highly divergent growth between domesticated, wild and hybrid strains of Atlantic salmon under hatchery conditions, with intermediate hybrid growth [24–27, 44]. Therefore, domesticated and wild populations of Atlantic salmon, like those used in the present study, represent a unique resource for studying genome-dosage effects. Unlike allopolyploids, which are genetic hybrids from two distinct species, Atlantic salmon are autoployploids, and the effect of genome dosage can be examined without the confounding effects of hybridity between distinct species [49].

## Conclusions

We have demonstrated that there is a dosage effect of ploidy interacting with parent-origin. The use of triploids has recently been marred by the contrasting results of their relative performance compared to diploids, however the present study indicates that the aquaculture industry should place more emphasis on maternal performance when creating triploid families to improve triploid growth performance. Although these experiments only included one domesticated strain, the observed results were consistent between the experiments and different families within. Further studies investigating the dosage effect of ploidy and parent-origin in environmental conditions where triploids are known to perform less well could provide further clarity on the genome-dosage effect and further elucidate the potential for parent-based selection and performance.

## Additional file

Additional file 1: Table S1. (summary data of experiment 1 with the outlier removed), **Table S2**. (family design of experiment 2), **Table S3.** (model selection for the random effects in experiment 2), and **Figure S1.** (an overview of experiment 1). (DOCX 35 kb)

## Abbreviations

AIC: Akaike information criterion
ANOVA: Analysis of variance
BIC: Bayesian information criterion
F1: First generation hybrids
IMR: Institute of Marine Research
LD: Light to dark ratio
PIT: Passive integrated transponders
SGR: Specific growth rate
WGD: Whole genome duplication.
