# Supplementary material for: Ploidy elicits a whole-genome dosage effect: growth of triploid Atlantic salmon is linked to the genetic origin of the second maternal chromosome set
Source: BMC Genet. 2017 Apr 11;18:34. doi: 10.1186/s12863-017-0502-x (PMC5387229; doi:10.1186/s12863-017-0502-x)
Supplement: Additional file 1: Table S1. — (summary data of experiment 1 with the outlier removed), Table S2. (family design of experiment 2), Table S3. (model selection for the random effects in experiment 2), and Figure S1. (an overview of experiment 1). (DOCX 35 kb) [file 12863_2017_502_MOESM1_ESM.docx]

**Additional file 1**

Table S1: Weight and specific growth rate (SGR) data for experiment 1 Phase II (end) with 3 outliers removed (471 PIT tagged individuals) and SGR data for experiment 1 Phase II with all PIT tagged individuals. Hybrid key: maternal x paternal; N*:* number of families or final number of fish in each group (including outliers were applicable); W (mass in grams) SE (standard error) SGR (specific growth rate); Percentage domesticated genome: the relative percentage of the individual’s genome coming from a domesticated parent.

|  |  |  | **Diploids** | | |  | **Triploids** | | |
| --- | --- | --- | --- | --- | --- | --- | --- | --- | --- |
| **Experiment** | **Group** | **N Families** | **% Domesticated Genome** | **Final N** | **Weight (+/- SE)** |  | **% Domesticated Genome** | **Final N** | **Weight (+/- SE)** |
| **1 – Phase II** | Wild | 1 | 0 | 57 | 94.12 (2.90) |  | 0 | 59 | 93.49 (3.24) |
| **(PIT tag)** | Wild x Domesticated | 1 | 50 | 59 | 138.46 (4.83) |  | 33.3 | 60 | 112.87 (3.87) |
|  | Domesticated x Wild | 1 | 50 | 58 | 143.02 (4.29) |  | 66.6 | 59 | 167.53 (3.80) |
|  | Domesticated | 1 | 100 | 60 | 198.23 (5.76) |  | 100 | 59 | 185.86 (4.79) |
|  |  | **N Families** | **% Domesticated Genome** | **Final N** | **SGR (+/- SE)** |  | **% Domesticated Genome** | **Final N** | **SGR (+/- SE)** |
| **1 – Phase II** | Wild | 1 | 0 | 57 | 0.27 (0.01) |  | 0 | 59 | 0.24 (0.01) |
| **(PIT tag)** | Wild x Domesticated | 1 | 50 | 59 | 0.27 (0.01) |  | 33.3 | 60 | 0.27 (0.01) |
|  | Domesticated x Wild | 1 | 50 | 58 | 0.37 (0.01) |  | 66.6 | 59 | 0.35 (0.01) |
|  | Domesticated | 1 | 100 | 60 | 0.31 (0.01) |  | 100 | 59 | 0.32 (0.01) |
| **1 – Phase II** | Wild | 1 | 0 | 59 | 0.29 (0.01) |  | 0 | 60 | 0.25 (0.01) |
| **(all** | Wild x Domesticated | 1 | 50 | 59 | 0.28 (0.01) |  | 33.3 | 60 | 0.27 (0.01) |
| **PIT tag)** | Domesticated x Wild | 1 | 50 | 58 | 0.37 (0.01) |  | 66.6 | 59 | 0.35 (0.01) |
|  | Domesticated | 1 | 100 | 60 | 0.31 (0.01) |  | 100 | 59 | 0.32 (0.01) |

Table S2: Family design and parental origin of the families used in experiment 2.

|  |  | Dam | | Sire | |
| --- | --- | --- | --- | --- | --- |
| Group | Family | Individual | Stock | Individual | Stock |
| Farm | 1 | 1 | Farm | 11 | Farm |
|  | 5 | 3 | Farm | 13 | Farm |
|  | 9 | 5 | Farm | 15 | Farm |
|  | 11 | 6 | Farm | 16 | Farm |
|  | 15 | 8 | Farm | 18 | Farm |
|  | 17 | 9 | Farm | 19 | Farm |
|  | 19 | 10 | Farm | 20 | Farm |
| Hybrid | 2 | 1 | Farm | 27 | Wild |
|  | 4 | 2 | Farm | 27 | Wild |
|  | 6 | 3 | Farm | 41 | Wild |
|  | 10 | 5 | Farm | 40 | Wild |
|  | 20 | 10 | Farm | 35 | Wild |

Table S3: Model selection of the random effect of the linear mixed effect model to investigate ploidy in experiment 2.

| A: Hybrid | | Random effects | | |  |  |  |  |  |  |  |
| --- | --- | --- | --- | --- | --- | --- | --- | --- | --- | --- | --- |
| N | Response | F | T | Df | AIC | BIC | logLik | Deviance | Chisq | Chi Df | P |
| 184 | Weight | x | x | 5 | 1250.5 | 1266.6 | -620.26 | 1240.5 | 0 | 1 | 1 |
|  |  | **x** |  | 4 | 1248.5 | 1261.4 | -620.26 | 1240.5 | 14.712 | 0 | <2e-16 |
|  |  |  | x | 4 | 1263.2 | 1276.1 | -627.61 | 1255.5 |  |  |  |
|  |  |  |  |  |  |  |  |  |  |  |  |
| B: Domesticated | | Random effects | | |  |  |  |  |  |  |  |
| N | Response | F | T | Df | AIC | BIC | logLik | Deviance | Chisq | Chi Df | P |
| 251 | Weight | x | x | 5 | 1787.4 | 1805.0 | -888.71 | 1777.4 | 0 | 1 | 1 |
|  |  | **x** |  | 4 | 1785.4 | 1799.5 | -888.71 | 1777.4 | 4.03 | 1 | 0.04 |
|  |  |  | x | 4 | 1789.4 | 1803.5 | -890.72 | 1781.4 |  |  |  |

F; family (random intercept). T; tank (random intercept). Df; degrees of freedom. AIC; Akaike information criterion. BIC; Bayesian information criterion. Loglik; log likelihood. Deviance; Chi.sq; value of the Chi square statistics. Chi.Df; the degrees of freedom for the test. P: P-value. The significant random effect structure is marked in bold.

Figure S1: Experimental design for experiment 1 indicating Phase I and Phase II. Initially 150 individuals from each group were reared in single strain tanks from until November 2012, when all individuals were sampled for biological measurements (end of Phase I). 480 individuals were taken randomly from the 1137, PIT tagged and mixed together into 3 tank replicates (160 per tank) (beginning Phase II). In June 2013 the experiment was terminated and all remaining fish (477) were sampled for biological measurements (end of Phase II).
